# Maternal Creatine Supplementation during Pregnancy Prevents Long-Term Changes in Diaphragm Muscle Structure and Function after Birth Asphyxia

**DOI:** 10.1371/journal.pone.0149840

**Published:** 2016-03-01

**Authors:** Domenic A. LaRosa, Stacey J. Ellery, Helena C. Parkington, Rod J. Snow, David W. Walker, Hayley Dickinson

**Affiliations:** 1 Ritchie Centre, Hudson Institute of Medical Research and Department of Obstetrics and Gynaecology, Monash University, Clayton, Victoria, Australia; 2 Department of Physiology, Monash University, Clayton, Victoria, Australia; 3 Centre for Physical Activity and Nutrition Research, School of Exercise and Nutrition Sciences, Deakin University, Burwood, Victoria, Australia; University of Louisville School of Medicine, UNITED STATES

## Abstract

Using a model of birth asphyxia, we previously reported significant structural and functional deficits in the diaphragm muscle in spiny mice, deficits that are prevented by supplementing the maternal diet with 5% creatine from mid-pregnancy. The long-term effects of this exposure are unknown. Pregnant spiny mice were fed control or 5% creatine-supplemented diet for the second half of pregnancy, and fetuses were delivered by caesarean section with or without 7.5 min of *in-utero* asphyxia. Surviving pups were raised by a cross-foster dam until 33±2 days of age when they were euthanized to obtain the diaphragm muscle for *ex-vivo* study of twitch tension and muscle fatigue, and for structural and enzymatic analyses. Functional analysis of the diaphragm revealed no differences in single twitch contractile parameters between any groups. However, muscle fatigue, induced by stimulation of diaphragm strips with a train of pulses (330ms train/sec, 40Hz) for 300sec, was significantly greater for asphyxia pups compared with controls (p<0.05), and this did not occur in diaphragms of creatine + asphyxia pups. Birth asphyxia resulted in a significant increase in the proportion of glycolytic, fast-twitch fibres and a reduction in oxidative capacity of Type I and IIb fibres in male offspring, as well as reduced cross-sectional area of all muscle fibre types (Type I, IIa, IIb/d) in both males and females at 33 days of age. None of these changes were observed in creatine + asphyxia animals. Thus, the changes in diaphragm fatigue and structure induced by birth asphyxia persist long-term but are prevented by maternal creatine supplementation.

## Introduction

Each year approximately 1–3 neonates in every 1000 suffer a period of oxygen (O_2_) deprivation at birth [1–3]. Asphyxia and hypoxia during labour or delivery is responsible for an estimated 1.2 million deaths each year, accounting for 29% of neonatal mortality [1–3]. Although birth asphyxia occurs all over the world, incidence and mortality rates are highest in remote areas, in low-income countries where healthcare is poor [4–6]. It can result from a variety of events including umbilical cord compression, protracted labour or placental abruption [1,3]. The profound hypoxia and resulting metabolic failure in the fetal tissues leads to the depletion of intracellular ATP and the generation of reactive oxygen and nitrogen species (ROS and RNS) [7,8]. This is particularly detrimental to tissues with high and fluctuating energy demands such as the brain and striated muscle, and in animal models of birth asphyxia this has also been shown to result in the induction of apoptosis and subsequent tissue damage or loss [9–12].

A major issue observed clinically in neonates after an asphyxic episode is respiratory insufficiency which can persist for many days, with the result that mechanical ventilation is often required [6,13,14]. Mechanical ventilation is known to result in deterioration of diaphragm muscle function (i.e. disuse atrophy) [15–18] with the result that patients often need to be ‘weaned’ off ventilatory support. Furthermore, neonates surviving birth asphyxia often have persisting respiratory problems, with an incidence as high as 86% reported [19,20]. Very little is known of the consequences of birth asphyxia on the diaphragm beyond the immediate neonatal period. However, with reports of increased incidence of respiratory conditions such as asthma and chronic obstructive pulmonary disease (COPD) after asphyxia at birth [21,22], the long-term effects of hypoxia *per se* on diaphragm at birth requires investigation. Recent work investigating the effects of other prenatal challenges such as intra-uterine infection or maternal glucocorticoid administration have reported significant structural and functional deficits in the diaphragm [23–26], highlighting the vulnerability of the respiratory musculature to disturbances of the intra-uterine environment in late fetal development.

Using a model of birth asphyxia in the precocial spiny mouse [10,11,27–31] we have reported that acute intra-partum asphyxia caused significant structural and functional damage in the diaphragm observed at 24 h after birth. This included significant atrophy of the three major muscle fibre types and a reduction in calcium activated force [10]. Furthermore, we reported that supplementing the maternal diet with 5% creatine monohydrate from mid-gestation to term prevented this acute, asphyxia-related injury to the diaphragm [10]. However, the long-term effects of birth asphyxia on respiratory muscle function in this model are not well understood, nor is it known if the apparent protective effects of creatine against the early effects of birth asphyxia translate to benefits at the juvenile and early adult stages of life. Therefore, we determined if birth asphyxia produced long-term deficits in diaphragm structure and function, and further, we determined if prenatal creatine treatment was able to prevent the occurrence of any such persisting change(s) long-term.

## Methods

### Ethics and animal husbandry

All experiments were approved in advance by Monash University Animal Ethics Committee, as well as the Australian Government’s Department of Primary Industries, and conducted in according to the Australian Code of Practice for the Care and Use of Animals for Scientific Purposes. The spiny mice used for this study were obtained from our own laboratory colony and housed, bred and mated as previously described [32].

### Diet

Pregnant dams were fed either a control diet of standard rat and mouse chow (2.16mg Creatine (Cr)/g) throughout pregnancy, or chow supplemented with 5% creatine monohydrate (32.44mg Cr/g) from day 20 of gestation (mid-pregnancy) to term (Specialty Feeds, Glen Forrest, Perth, Australia; Creatine, Sigma). Water was freely available and animals were housed in family groups.

### Experimental groups

Animals born from control fed dams were termed the asphyxia group, and those from Cr fed dams were Cr+asphyxia. The control groups consisted of pups that were delivered by caesarean section without birth asphyxia from either control fed (c-section) or creatine-fed dams (creatine).

### Birth Asphyxia

The model of birth asphyxia used in this study has been extensively described and characterised by us [10,27,30,31]. Briefly, one day before term (term 39 days) the pregnant dam was killed by cervical dislocation, a midline abdominal incision was made and the entire uterus removed after tying off the uterine horns at the ovary and cervix with surgical silk. The uterus, with the fetuses inside, was then placed in a saline bath at 37°C during which progressive fetal hypoxia, hypercapnia, and acidemia developed [10]. After 7.5 min, the fetuses were quickly expelled from the uterus, their mouths cleared of fetal membranes, and the chest gently palpated using a moist cotton tip to stimulate breathing. The placenta was removed after approximately 20 min and pups were then allowed to recover for approximately 1 h in warmed sawdust obtained from the cage of the dam allocated to be the cross-foster mother.

Pups were cross-fostered to a lactating dam at approximately 1 h post-asphyxia and the cage was left undisturbed for 72 h. For all pups, the cross-foster females had been maintained on a normal diet and had given birth in the previous 24 h, and her own pups removed (killed, and when possible used as part of other studies) to ensure adequate lactation and to maximise neonatal care. All pups were then nursed and maintained by the cross-foster dam until 33±2 days of age (n = 10 per group, 5 males, 5 females).

### Ex vivo muscle function

This experiment was modified from one previously reported [33]. After 33±2 days, animals were humanely killed by cervical dislocation. The entire diaphragm was dissected with the last rib still attached and placed in ice-cold Krebs solution (120mM NaCl, 4.7 mM KCl, 1.2 mM MgSO_4_, 1.2 mM KH_2_PO_4_, 2.5 mM CaCl_2_, 25 mM NaHCO_3_, 11.1 mM glucose) and pre-bubbled with carbogen (95% O_2_ and 5% CO_2_) The diaphragm was cut in half, with one half immediately snap frozen in precooled isopentane and stored at -80°C for histological analyses. The other half was mounted in an *in vitro* organ bath system to assess contractile function. The diaphragm was anchored by the rib to a tissue holder and attached to a force transducer via the central tendon using surgical silk.

The organ bath contained Krebs solution maintained at 34°C and bubbled with carbogen (95% O_2_ and 5% CO_2_). During the initial equilibration period, the hemi-diaphragm was gently stretched using a venier adjuster to obtain optimum length for the production of maximal twitch force. Muscle contractions were induced by electrical stimulation using a platinum ring electrode. Stimuli (0.2 ms duration) to induce single twitch contractions were delivered at 1 pulse per second at 1.2–1.5x maximum voltage. Direct stimulation was chosen to ensure results were not confounded by changes in innervation. Fatigue was induced by repetitive tetanic stimulation using a train of pulses (0.2 ms, 40 Hz, 330 ms train duration) repeated at 1 train/sec for 300 sec; the fatigue index was derived from the relative decrease in tetanic force after 50 and 300 sec of repeated stimulation. All twitch tension and fatigue recordings were stored digitally using an A-D converter, and analysed later using Labchart software (Version 8, AD Instruments, Sydney, Australia).

As the entire hemidiaphragm was used in these functional studies, samples contained individual fibres of a range of different lengths. As such, cross-sectional area (CSA) could not be accurately determined for the purposes of normalisation of force measurements, which is convention when using strips of muscle to assess contractile function. Therefore, force measurements were expressed relative to muscle weight (g(force)/mg(muscle), which allowed comparison between the four groups.

### Histochemistry

**Fig 1:**
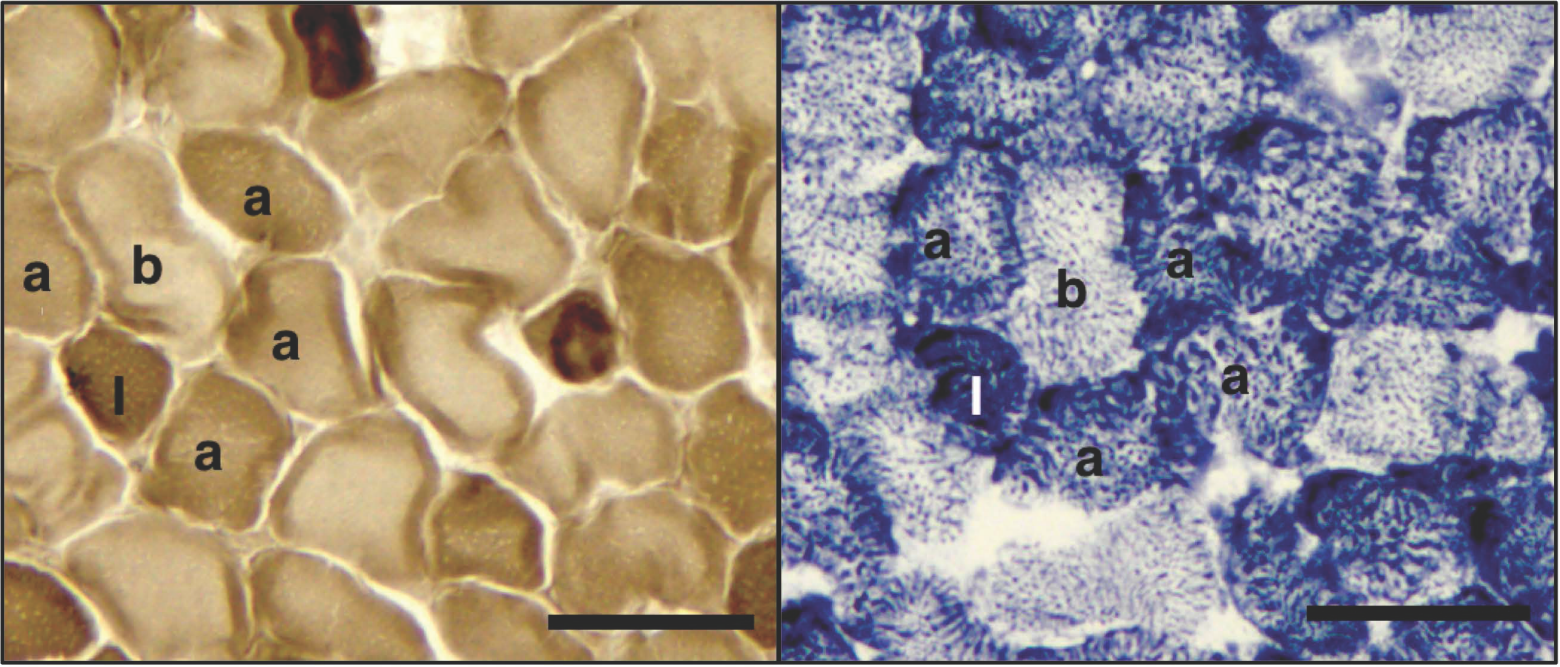
Representative images of (A) ATPase and (B) SDH stained diaphragm sections. Serial sections of the diaphragm from a 33 day old spiny mouse are shown. For ATPase, after a pre-incubation at pH 4.6, Type I fibres stain dark (I), Type IIa fibres stain intermediate (a), and Type IIb/d fibres stain pale (b). After SDH staining, Type I fibres stain intensely (I), Type IIa fibres stain intermediately (a), and Type IIb/d fibres stain lightly (b). Black line is 50μm for all panels.

Transverse, 10μm sections were cut from frozen diaphragm samples using a cryostat microtome (Leica) and adhered to Superfrost® Plus slides (Menzel-Gläser). Myofibrillar ATPase staining was employed to differentiate Type I (slow-oxidative), Type IIa (fast-oxidative) and Type IIb/IId (fast-glycolytic) fibers as previously described [10,34] with the following modifications: sections were pre-incubated in acetate buffer (100mM sodium acetate and 50mM HCl, pH adjusted to 4.6 using NaOH) for 12 min (pH 4.6), then incubated for 30 min in ATPase solution (20mM CaCl_2_, 20mM sodium barbitone, 3mM ATP, pH 9.4) at 37°C. Sections were then rinsed in distilled deionized water (ddH_2_0, 2 min), immersed in 1% CaCl_2_ (2 min), rinsed (2 min; dH_2_O), immersed in 2% CoCl_2_ (3 min), rinsed (2 min; ddH_2_O), immersed in 5% ammonium persulphate (30 sec) and rinsed again (2 min: ddH2O) before sections were dehydrated with ethanol (70%, 2 x 100%; 2 min each). Sections were then mounted using DPX mounting medium and allowed to dry for 24 h. Using this protocol, Type I fibers stain dark brown, Type IIa stain intermediate, and Type IIb/d remain pale. A representative image of the ATPase staining is shown in Fig 1A. The distribution and cross-sectional area (μm^2^) of individual fibre types were determined for ~100 fibres per transverse section of diaphragm from all animals in each group (n = 10 per group, 5 males, 5 females) using computer software (Image J, Research Services Branch, National Institute of Health, Bethesda, MA, USA).

Succinate dehydrogenase (SDH) abundance was used to determine the oxidative capacity of individual diaphragm fibres, using sections adjacent to those used for myofibrillar ATPase. The SDH protocol was modified from a previously described method [35]. Briefly, sections were incubated in 100mM phosphate buffer (pH 7.6), 50mM succinate, 5mM EDTA, 1.5mM nitro-blue tetrazolium (NBT), 1mM sodium azide and 0.2mM phenazine at 37°C for 15 min. Sections were then submerged sequentially in 20%, 60% and 90% aqueous acetone before being rinsed for 2 min in ddH_2_O, and cover-slipped using DPX. Incubation without succinate was carried out to determine non-specific staining. Densitometric analysis using Image J software was used to categorise the oxidative capacity of individual fibres based on their staining intensity, with a greater optical density indicating a higher abundance of this enzyme (LaRosa *et al*., 2012). Results were expressed in Arbitrary Units (AU). As these were serial sections to those stained for ATPase activity, the ATPase stained images were used as a reference and the same fibres were identified on the SDH stained slides. This allowed the mean oxidative capacity for each fibre type to be determined, which was achieved by measuring n = 20 fibres of each fibre type, for each animal (n = 10 per group, 5 males, 5 females). A representative image of SDH staining is shown in Fig 1B, indicating the same fibres in both ATPase and SDH images.

All slides were coded before any of the assessments described above took place, so that the assessor was ‘blinded’ with respect to birth group, offspring sex, and maternal diet treatment.

### Statistical analysis

Survival data were analysed with a Chi-square test for independence. All other data are presented as mean ± standard error of the mean (SEM). Muscle structure and function parameters for males and females were assessed separately using a 2-way ANOVA, assessing the effects of birth type (p_BIRTH_) and maternal diet (p_DIET_), followed by post hoc analysis with Tukey’s multiple comparisons where differences in ANOVA were detected using statistical software (Prism 6, Graphpad software Inc.^TM^, USA). Postnatal growth results were assessed using a 3-way ANOVA, assessing the effects of postnatal age (p_AGE_), birth type (p_BIRTH_) and maternal diet (p_DIET_). Where only one set of *p* values are reported, results were the same for males and females. Numbers indicate the number of pups per treatment group, with only 1 male and 1 female obtained from any one litter. Statistical significance was accepted when *p* < 0.05.

## Results

### Survival and postnatal growth

Survival rates in the immediate neonatal period (1 h) after our birth asphyxia protocol for this study are summarised in Table 1, and were similar to those observed in a previous study using this model [27]. The birth asphyxia protocol resulted in an overall survival rate of 59%, but this was lower for males (52%) than for females (69%). Maternal creatine supplementation significantly increased neonatal survival rate overall by 17% (males, 19%, p<0.001; females, 12%, p<0.01). The surviving offspring of each treatment group grew at similar rates over the 31–33 days regardless of sex (Table 2).

**Table 1:** The number of dams pups, and the survival rates for each treatment group immediately after birth.

Treatment	Dams	Sex	Fetuses	Alive	Dead	Survival Rate
**C-Section**	24	Male	38	38	0	100%
		Female	27	27	0	100%
**Asphyxia**	39	Male	67	35	22	52%
		Female	48	33	15	69%
**Creatine**	17	Male	21	21	0	100%
		Female	25	25	0	100%
**Cr +**	23	Male	38	27	11	71%^a^
**Asphyxia**		Female	41	33	8	81%^b^

^*a*^ *p = 0*.*0004*, between male asphyxia groups

^*b*^ *p = 0*.*005*, between female asphyxia groups

**Table 2:** Postnatal growth for each treatment group from birth to 33±2 days. 3-way repeated measures ANOVA showed a significant effect of age (p<0.05) in all treatment groups. No significant effects of birth or diet were found (p>0.05). Values are means ± SEM; n ≥6/group.

Treatment	Sex	1	7	14	21	28	33±2
**C-Section**	M	5.4±0.1	8.9± 0.3	11.6±0.3	16.3±0.7	22.7±0.6	26.1±1.4
	F	4.9±0.2	8.6±0.3	11.2±0.3	15.0±0.3	21.1±0.5	24.1±0.9
**Asphyxia**	M	5.0±0.1	7.9±0.4	10.6±0.6	15.0±0.8	20.2±1.0	26.9±0.8
	F	5.0±0.1	8.2±0.3	11.5±0.2	16.2±0.4	21.4±0.8	23.8±2.1
**Creatine**	M	5.1±0.1	8.2±0.3	11.5±0.5	15.0±0.8	21.3±1.3	23.1±1.1
	F	4.9±0.1	8.6±0.4	11.1±0.4	15.2±0.7	21.6±1.0	23.5±1.3
**Cr +**	M	5.2±0.1	9.1±0.5	12.7±0.9	17.2±1.5	23.4±1.5	25.1±1.5
**Asphyxia**	F	4.9±0.1	8.4±0.5	12.1±0.5	17.1±1.0	22.1±0.8	26.0±1.0

### Diaphragm muscle fibre morphology

#### Proportion of fibre types

**Fig 2:**
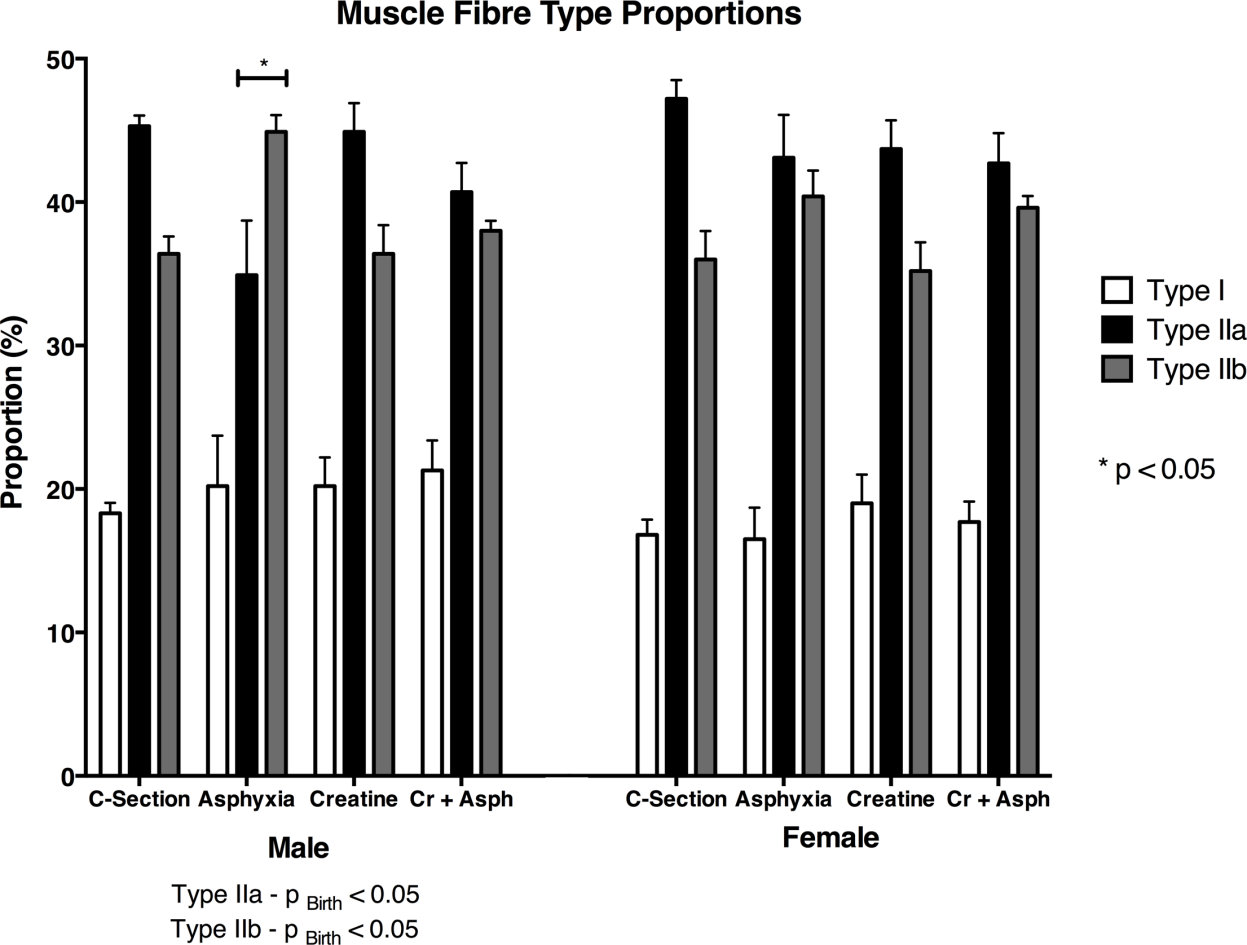
Mean proportions of the different fibre types present in the spiny mouse diaphragm at 33 days of age for the four treatment groups. Values are means ± SEM; n = 5/group. * indicates significant difference to all other groups.

The proportions of each fibre type present in the diaphragm, for each treatment group at 31–33 days of age are shown in Fig 2 (S1 Table). There were no differences between any of the treatment groups in the proportion of Type I (slow-oxidative) fibres present in the diaphragm, and there were no significant differences between the sexes. In male offspring, there was a significant difference in the relative numbers of Type IIa and IIb fibres between groups (p<0.05, Fig 2, S1 Table). In the c-section pups there was a greater number of IIa (fast-oxidative) fibres compared to IIb (fast-glycolytic) fibres, whereas in the diaphragm of male pups from asphyxia group there was a greater number of IIb fibres compared to IIa fibres (Fig 2, S1 Table). This alteration (or, ‘switch’) in the relative number of IIa and IIb fibres was not present in the diaphragms of pups from the creatine or Cr+asphyxia groups (Fig 2, S1 Table).

#### Muscle fibre size

**Fig 3:**
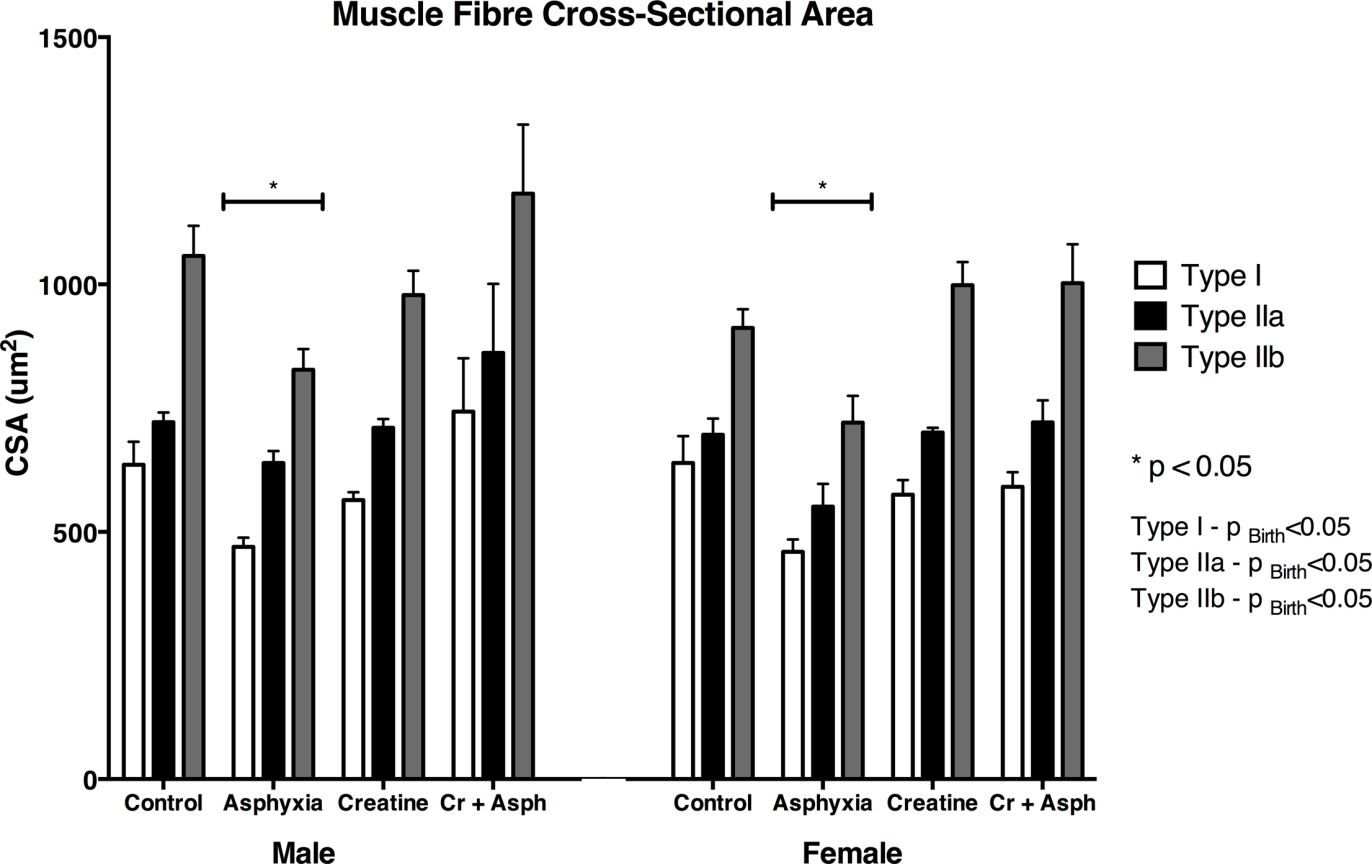
Mean cross-sectional area of the different fibre types in the spiny mouse diaphragm at 33 days of age for the four treatment groups. Values are means ± SEM; n = 5/group. * indicates significant difference to all other groups.

Fibre CSA for each fibre type in the diaphragm at 31–33 days of age is shown in Fig 3 (S2 Table). Birth asphyxia was associated with a significant reduction of CSA for all three fibre types in both males and females (Fig 3, S2 Table; p<0.05), and these changes were not observed in diaphragms obtained from animals in the Cr+ asphyxia or the creatine group (Fig 3, S2 Table).

### Oxidative capacity

**Fig 4:**
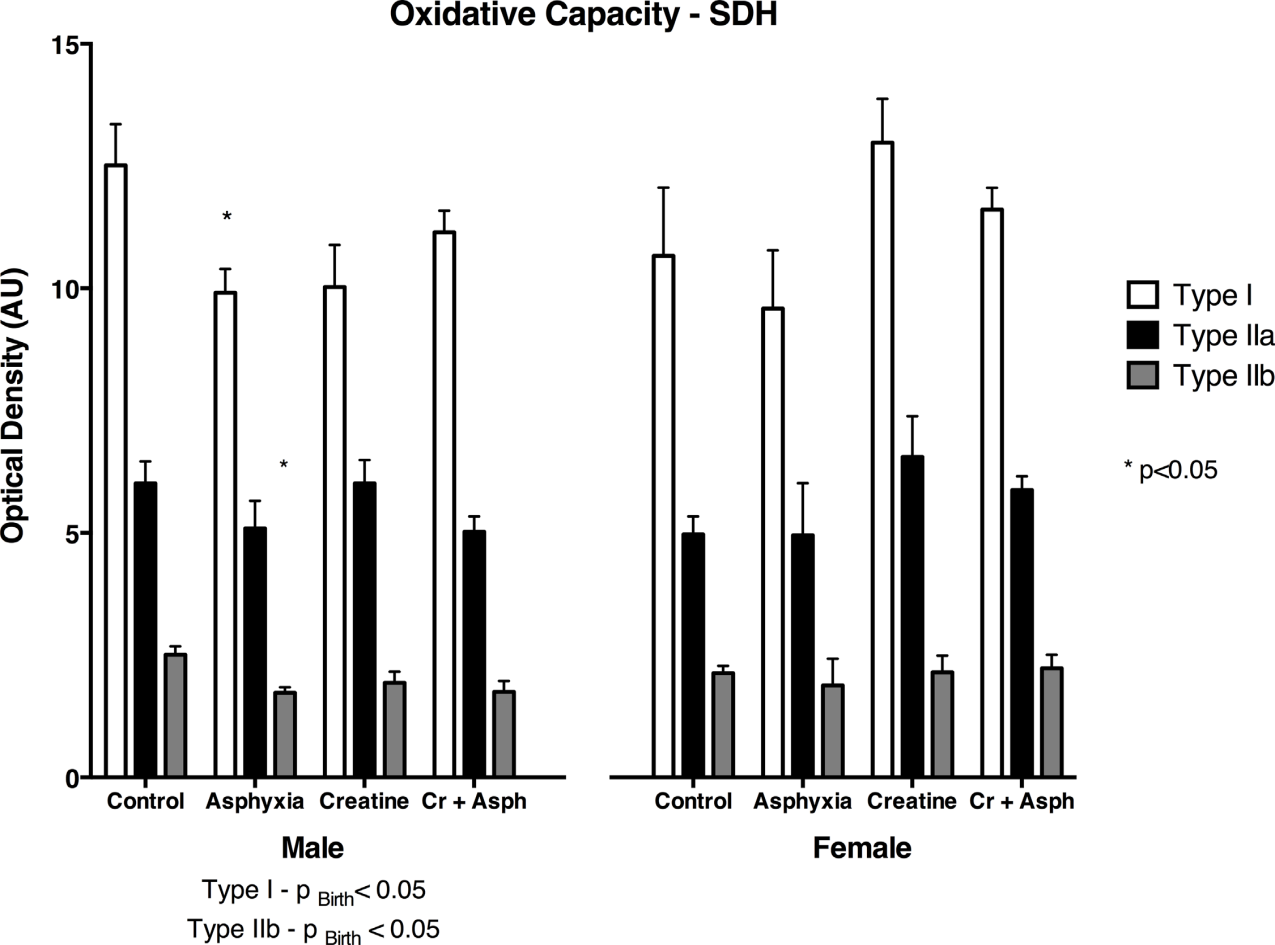
Summary of SDH abundance of all three muscle fibre types in the diaphragm of spiny mice from our four treatment groups at 33 days of age. Values are means ± SEM; n = 5/group. * indicates significant difference to all other groups.

SDH abundance was used to quantify the oxidative capacity of each fibre type using densitometry analysis (Fig 4, S3 Table). A significant reduction in SDH abundance in Type I and Type IIb fibres occurred in male asphyxia offspring only (p_BIRTH_<0.05, p_INT_>0.05) when compared to the c-section control group. Furthermore, these changes were not present in the male survivors of birth asphyxia where the mother had received the creatine diet (Fig 4, S3 Table).

### Ex vivo muscle function

For a single twitch at 33 days of postnatal age, the mode of birth (c-section vs asphyxia) or maternal diet (control vs 5% creatine) had no effect on diaphragm peak twitch tension, time to peak twitch tension or twitch half-relaxation time (Table 3).

**Table 3:** E*x-vivo* diaphragm muscle function at 31–33 days of age. 2-Way ANOVA found no significant differences for peak twitch tension, time to peak twitch tension or time to ½ relaxation tension (p>0.05) between the four treatment groups. No effect of sex was observed for any of the above parameters (p>0.05). Values are means ± SEM; n = 5/group.

		C-Section	Asphyxia	Creatine	Creatine + Asphyxia	P Value
**Peak Twitch Tension (g/mg)**	Male	0.067 ± 0.009	0.066 ± 0.009	0.085 ± 0.009	0.095 ± 0.020	p_Birth_ NS p_Diet_ NS
	Female	0.078 ± 0.011	0.102 ± 0.012	0.102 ± 0.012	0.065 ± 0.016	p_Int_ NS
**Time to Peak Twitch Tension (sec)**	Male	0.022 ± 0.002	0.023 ± 0.002	0.022 ± 0.002	0.028 ± 0.003	p_Birth_ NS p_Diet_ NS
	Female	0.024 ± 0.002	0.026 ± 0.002	0.027 ± 0.003	0.025 ± 0.002	p_Int_ NS
**Time to ½ Relaxation (sec)**	Male	0.028 ± 0.002	0.026 ± 0.004	0.032 ± 0.003	0.026 ± 0.002	p_Birth_ NS p_Diet_ NS
	Female	0.032 ± 0.002	0.025 ± 0.006	0.028 ± 0.002	0.031 ± 0.003	p_Int_ NS

**Fig 5:**
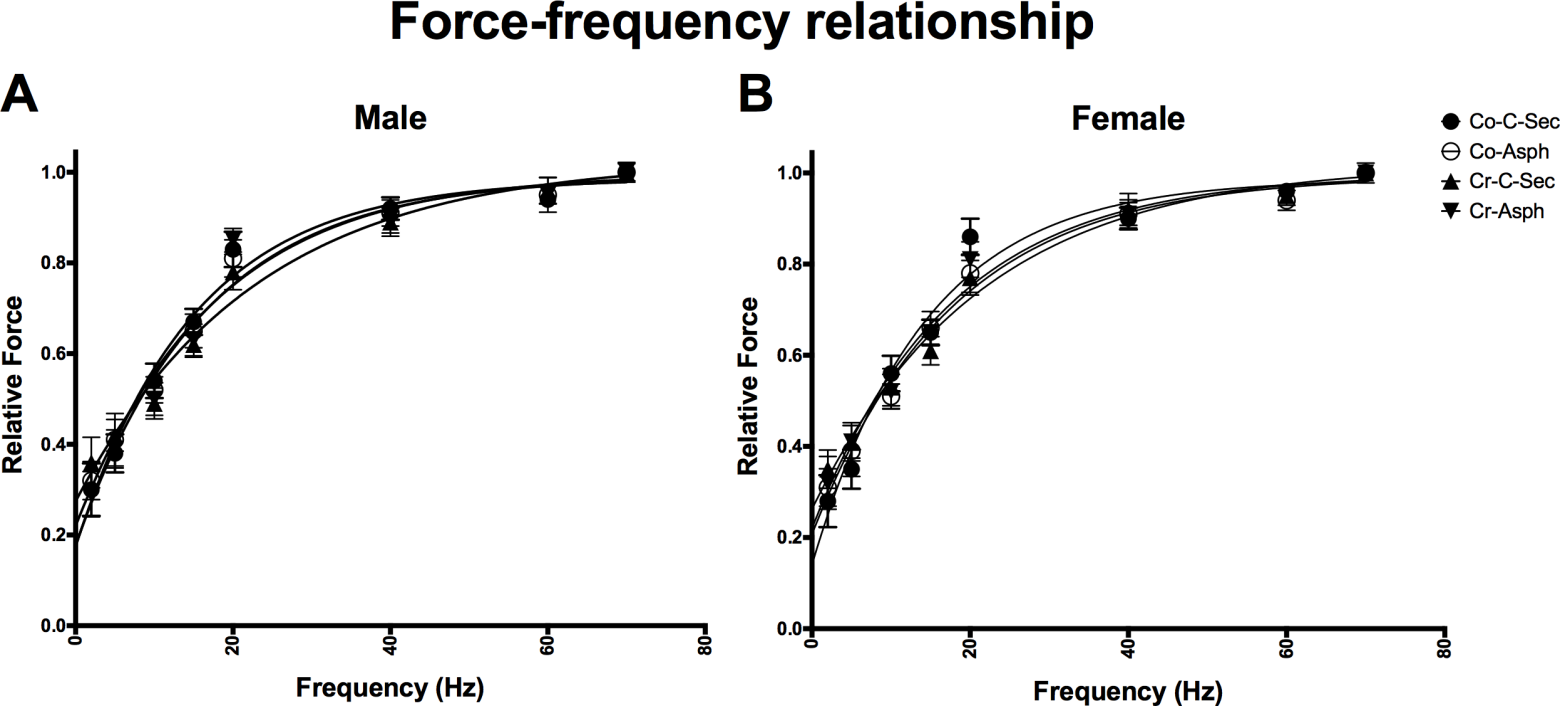
Normalised force-frequency relationships of the diaphragm for (A) male and (B) female spiny mice at 33 ± 2 days of age. Values are means ± SEM; n = 5/group.

**Fig 6:**
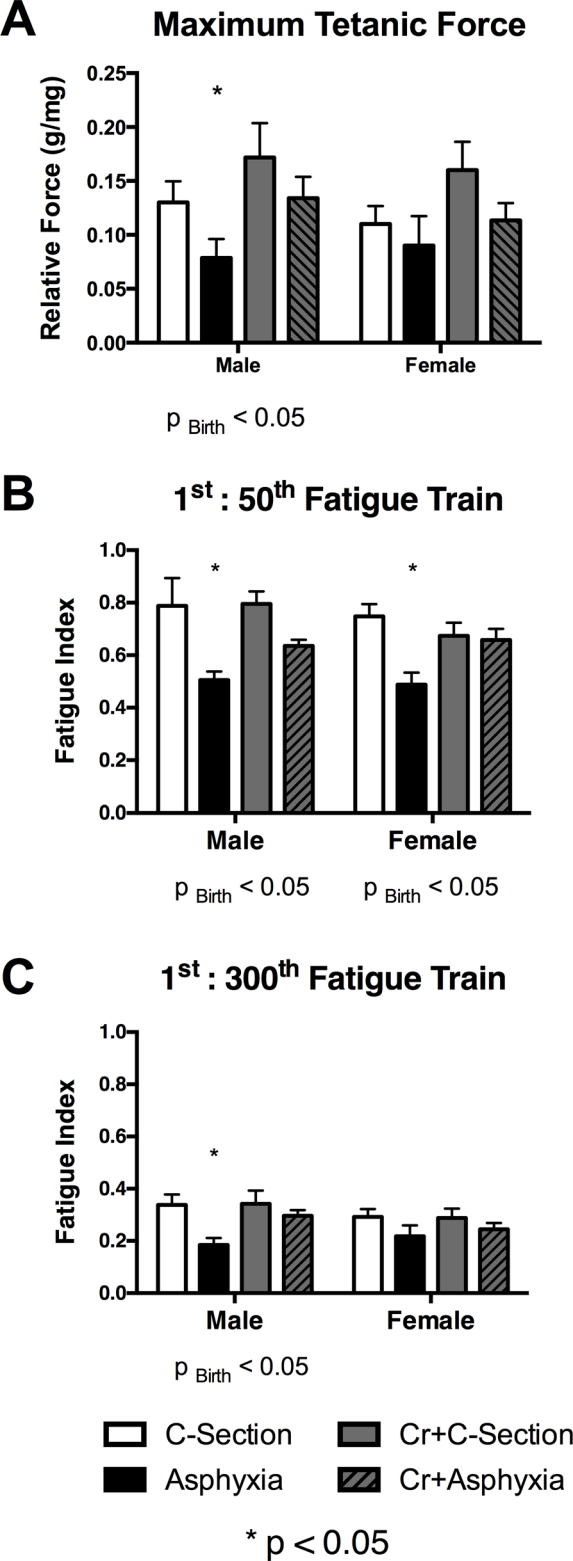
Summarises the (A) maximum tetanic force and the reduction in force during a fatigue train experiment at (B) the 50^th^ contraction and (C) the 300^th^ contraction of a fatigue train experiment in diaphragm muscle taken from our four treatment groups. Values are means ± SEM; n = 5/group. * indicates significant difference to all other groups.

The force-frequency relationship of the diaphragm was plotted and there were no significant differences between the groups (Fig 5, S4 Table). Relative maximum tetanic force, obtained at the conclusion of the force frequency relationship, was significantly reduced in diaphragm muscle of males from the birth asphyxia group (p_BIRTH_<0.05, p_INT_>0.05), a difference not evident in females. Furthermore, this reduction was prevented by maternal creatine supplementation (Fig 6A, S5 Table).

**Fig 7:**
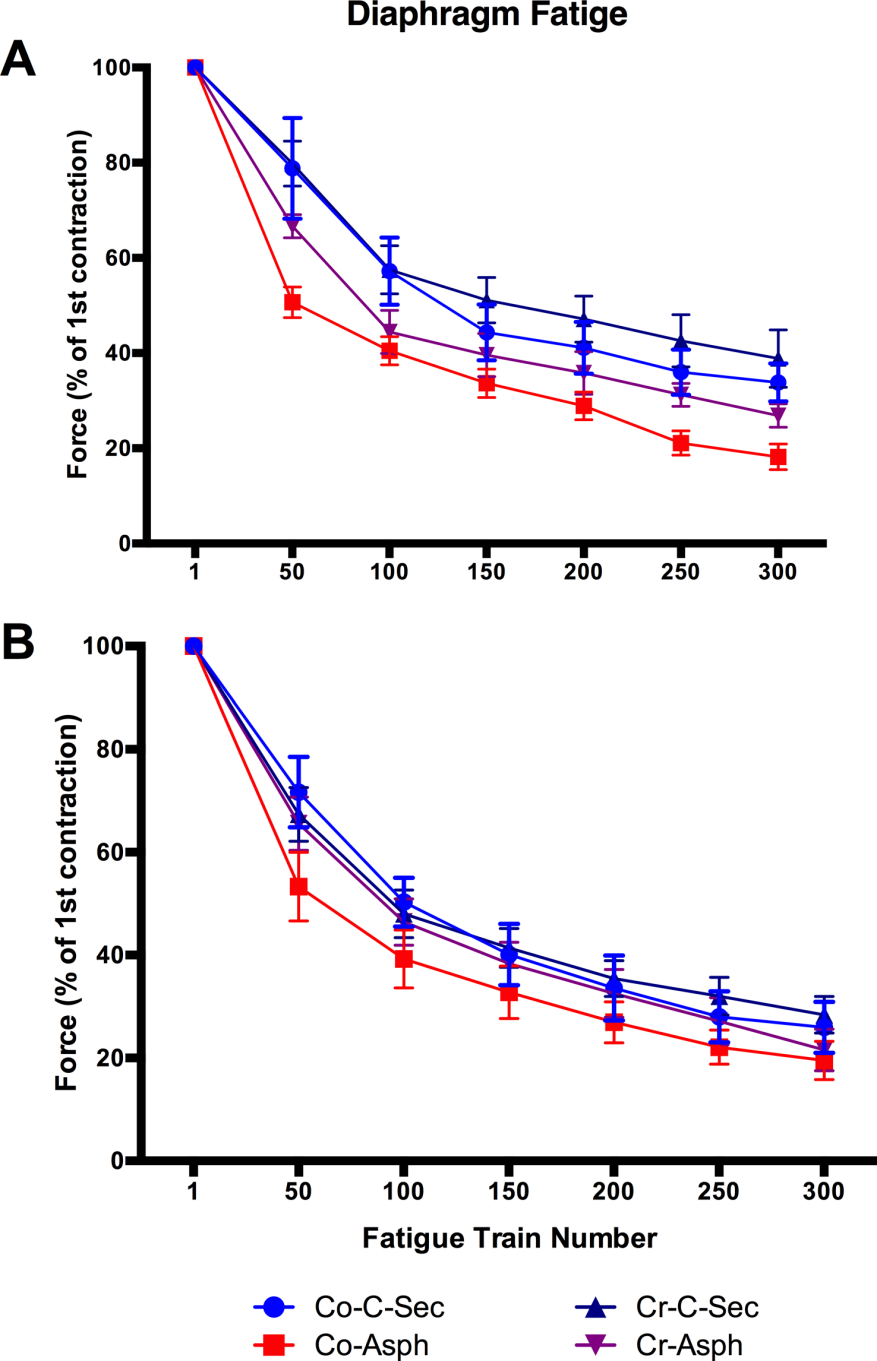
Illustrates the decay in diaphragm contractile force over a train of 300 contractions in (A) male B) female spiny mice from our four treatment groups. Values are means ± SEM; n = 5/group.

Fatigue resistance was assessed from the rate of decay of force during a sequence of 300 x 330 ms tetanic contractions induced at 1/sec over 5 min. Tetanic force decreased progressively throughout this series, but significantly more so for diaphragms from male and female offspring when the relative decrease of tetanic force was determined at the 50^th^ contraction (p_BIRTH_<0.05, p_INT_>0.05; Fig 6B, S6 Table). At the 300^th^ contraction, the asphyxia group exhibited a significantly higher degree of fatigue than the c-section group, but this reduction was only significant in males (p_BIRTH_<0.05, p_INT_>0.05; Fig 6C, S7 Table). The decay in force over the fatigue train experiment for all treatment groups is shown in Fig 6 and demonstrates that the rate of decay in both male (Fig 6A, S8 Table) and female (Fig 6B, S8 Table) asphyxia diaphragms was highest in over the first 50 contractions. The degree of fatigue in the asphyxia was then not different from their c-section counterparts, whereas the diaphragm from male offspring continued to fatigue at a higher rate (Fig 7). This increased fatigue (i.e., reduced fatigue resistance) was not evident in the creatine + birth asphyxia group at any time point in the series of tetanic contractions.

## Discussion

We have previously reported significant structural changes and functional deficits in the newborn spiny mouse diaphragm at 24 h after birth asphyxia, which did not occur if the mother had been given a diet supplemented with 5% creatine during the latter half of pregnancy [10]. The present study demonstrates that some structural and functional compromise of the diaphragm persists until at least 31–35 days after birth asphyxia. We observe a reduction in fibre size, changes in the relative number of Type IIa and IIb fibres, reduction in maximal force production, and decreased fatigue resistance. These alterations were not observed when the mother had consumed a diet supplemented with creatine over the second half of pregnancy. This study also confirms that supplementing the maternal diet with creatine improves neonatal survival in this model of birth asphyxia, a finding consistent with our previous investigations in this species [10,11,27]. Furthermore, prenatal creatine supplementation alone had no significant effects on postnatal growth or diaphragm muscle structure or function.

That birth asphyxia results in respiratory distress in the immediate neonatal period has been reported [36–38], with affected newborns requiring varying degrees of mechanical ventilatory support. However, it remains unclear to what extent this is due to hypoxia-induced lung damage, central decrease of respiratory drive due to damage to the brainstem, or to a direct compromise of the contractile function of the respiratory muscles. The results of our previous study in the spiny mouse showed that the diaphragm is directly affected by birth asphyxia [10]. The results of the present study indicate that some of these early (i.e. at 24 h) changes in diaphragm structure and function persist in postnatal life, as shown by reduced capacity of the isolated diaphragm to perform forced (tetanic) contractions and to endure fatigue. Moreover, the diaphragm of male survivors is more severely affected than females.

Other adverse events occurring during pregnancy also have significant structural and functional consequences for the respiratory musculature. For example, intrauterine inflammation, induced by intra-amniotic injection of LPS, caused significant reductions in peak twitch tension and maximum tetanic force in the preterm sheep diaphragm [25]. Another study by the same group found that maternal glucocorticoid administration, a standard clinical practice in anticipation of impending preterm birth, resulted in reduced peak twitch tension, lower post-fatigue force and an altered force-frequency relationship in preterm rat pups [26]. While it is a commonly held view that skeletal muscle is highly adaptable (i.e., ‘plastic’) and has a high capacity for repair [39–43], the results of the present study indicate that this is not so the case for the diaphragm after asphyxia at birth, with the significant structural and functional deficits observed 24 h after birth asphyxia [10] persisting to at least 33 d of age.

The asphyxia-induced changes in diaphragm function observed in this study are consistent with the structural changes. The increased fatigue is consistent with the increased proportion of Type IIb fibres in the control diet birth asphyxia group, because these glycolytic, fast-twitch fibres are known to fatigue more quickly than the oxidative, Type IIa fibres [44,45]. Taken together with the significant reduction in the oxidative capacity, as assessed by reduced SDH abundance, of Type I and Type IIb fibres, the decrease in fatigue resistance of the diaphragm after birth asphyxia are explicable if there is a greater reliance on Type IIb/d fibres during forced contractions, which were also found to have a lower oxidative capacity in male birth asphyxia offspring.

The changes in the proportion of fibre types in the diaphragm of male birth asphyxia offspring may be due to perturbation of normal postnatal development of the diaphragm. Previous studies investigating pre- and postnatal development of the sheep diaphragm have reported a significant increase in the relative expression of MHC-IIa in the early postnatal period [46]. Furthermore, we have found that the spiny mouse diaphragm undergoes significant changes in fibre type proportions in the immediate neonatal period, with a significant increase in the number of Type I fibres and a switch in fast fibre type predominance from Type IIb in the late gestation to Type IIa by postnatal day 3 [Cannata, LaRosa, Dickinson, Walker, unpublished data]. Therefore, exposure to a hypoxic episode, induced by birth asphyxia, may disrupt this normal developmental process, particularly in male offspring, however the mechanisms involved in this disruption require further investigation.

The sexual dimorphism evident in neonatal survival following birth asphyxia was also present in the increased severity of structural and functional diaphragm damage in the male offspring at 31–33 days of age. Birth asphyxia-related mortality and morbidity is significantly higher in human male infants [47–49], and has been attributed to inherent differences in placental structure and function [50,51]. Steroid hormones also influence muscle cell responses to cellular injury; e.g. testosterone has been reported to augment inflammation and apoptosis by inhibiting the activation of nitric-oxide synthase, which is known to activate anti-inflammatory and anti-apoptotic pathways, and these pathways are up-regulated by oestrogen [52–54] perhaps accounting for the increased susceptibility to exercise induced muscle damage in males in comparison to females. The developmental profile of testosterone in the spiny mouse has not yet been reported, therefore this remains to be elucidated.

A limitation of the present study is that intact respiratory function was not assessed. Preliminary studies using plethysmography at rest have not found any major effects of birth asphyxia on resting ventilation at 31–33 days [LaRosa, unpublished observations]. The finding that maximum tetanic force and resistance to fatigue are both reduced, leads to the prediction that ventilation under load or when increased respiratory effort is required, may be compromised. Additionally, the persistent changes in diaphragm structure and function identified in this study may have significant implications should survivors of birth asphyxia go on to develop obstructive and restrictive respiratory disorders, as such conditions require increased mechanical work by the diaphragm. Studies have reported a higher incidence of asthma in pre-school aged children who suffered a hypoxic event in the perinatal period [21], and children afflicted with cerebral palsy, a common outcome of birth asphyxia, are more likely to develop respiratory conditions such chronic obstructive pulmonary disease (COPD) [22]. This study has shown that a hypoxic episode at birth, without producing obvious neurological damage results in diaphragm muscle damage, which could act to reduce the respiratory and athletic capacity of survivors of mild birth asphyxia. Therefore the role of the diaphragm in the respiratory morbidities observed in human infants exposed to birth asphyxia should be further elucidated.

Finally, the results of this study illustrate that supplementing the maternal diet with creatine prior to a hypoxic event at birth can largely prevent long-term deficits in respiratory muscle structure and function. While not assessed in this study, it is hypothesised that by increasing cellular Cr/PCr levels, cellular ATP turnover is maintained during the period of birth asphyxia, thus reducing or preventing induction of cellular injury pathways. This may also be augmented by the inherent antioxidant properties of creatine [55,56]. It is also important to note that, by itself, the creatine treatment had no significant effects on postnatal outcomes, or on the relative size and numbers of muscle fibres contained in the diaphragm. Therefore, the findings of this study strengthen the case for the clinical translation of creatine in human obstetrics, as argued elsewhere [57,58].

## Supporting Information

S1 Table Muscle fibre type proportions in the spiny mouse diaphragm at 33 days of age for the four treatment groups. (PDF)

S2 Table Cross-sectional area of the different fibre types in the spiny mouse diaphragm at 33 days of age for the four treatment groups. (PDF)

S3 Table SDH abundance of all three muscle fibre types in the diaphragm of spiny mice from our four treatment groups at 33 days of age. (PDF)

S4 Table Normalised force-frequency relationships of the diaphragm for male and female spiny mice at 33 of age. (PDF)

S5 Table Maximum tetanic force produced by diaphragm muscle taken from our four treatment groups. (PDF)

S6 Table Force at the 50^th^ contraction of a fatigue train experiment in diaphragm muscle taken from our four treatment groups. (PDF)

S7 Table Force at the 300^th^ contraction of a fatigue train experiment in diaphragm muscle taken from our four treatment groups. (PDF)

S8 Table Decay in diaphragm contractile force over a train of 300 contractions in male female spiny mice from our four treatment groups. (PDF)
