# Supplementary material for: Maternal Creatine Supplementation during Pregnancy Prevents Long-Term Changes in Diaphragm Muscle Structure and Function after Birth Asphyxia
Source: PLoS One. 2016 Mar 1;11(3):e0149840. doi: 10.1371/journal.pone.0149840 (PMC4773130; doi:10.1371/journal.pone.0149840)
Supplement: S1 Table — (PDF) [file pone.0149840.s001.pdf]

		Type I			Type IIa			Type IIb		
		Mean	SEM	N	Mean	SEM	N	Mean	SEM	N
C-Section	Male	18.3	0.74	5	45.3	0.73	5	36.4	1.2	5
Asphyxia		20.2	3.52	5	34.9	3.82	5	44.9	1.17	5
Creatine		20.2	2	5	44.9	2	5	36.4	2	5
Cr+Asphyxia		21.3	2.09	5	40.7	2.03	5	38	0.7	5
C-Section	Female	16.8	1.07	5	47.2	1.31	5	36	1.98	5
Asphyxia		16.5	2.2	5	43.1	2.98	5	40.4	1.8	5
Creatine		19	2	5	43.7	2	5	35.2	2	5
Cr+Asphyxia		17.7	1.42	5	42.7	2.11	5	39.6	0.82	5
