# Supplementary material for: Maternal Creatine Supplementation during Pregnancy Prevents Long-Term Changes in Diaphragm Muscle Structure and Function after Birth Asphyxia
Source: PLoS One. 2016 Mar 1;11(3):e0149840. doi: 10.1371/journal.pone.0149840 (PMC4773130; doi:10.1371/journal.pone.0149840)
Supplement: S2 Table — (PDF) [file pone.0149840.s002.pdf]

		Type I				Type IIa				Type IIb		
		Mean	SEM	N		Mean	SEM	N		Mean	SEM	N
C-Section	Male	636.0	45.9	5	Male	721.4	19.6	5	Male	1057.4	61.3	5
Asphyxia		469.8	18.4	5		639.3	24.5	5		827.5	41.9	5
Creatine		564.3	16.2	5		709.8	17.9	5		978.5	48.8	5
Cr+Asphyxia		742.8	107.9	5		861.5	139.7	5		1183.5	139.9	5
C-Section	Female	639.3	53.9	5	Female	696.0	32.8	5	Female	912.0	37.7	5
Asphyxia		459.6	25.1	5		551.0	46.3	5		720.4	54.3	5
Creatine		575.4	29.6	5		700.6	9.5	5		998.6	46.9	5
Cr+Asphyxia		591.0	29.9	5		720.8	44.6	5		1002.4	78.9	5
