# Supplementary material for: Maternal Creatine Supplementation during Pregnancy Prevents Long-Term Changes in Diaphragm Muscle Structure and Function after Birth Asphyxia
Source: PLoS One. 2016 Mar 1;11(3):e0149840. doi: 10.1371/journal.pone.0149840 (PMC4773130; doi:10.1371/journal.pone.0149840)
Supplement: S3 Table — (PDF) [file pone.0149840.s003.pdf]

		Type I			Type IIa			Type IIb		
Group	Sex	Mean	SEM	N	Mean	SEM	N	Mean	SEM	N
**C-Section**	**Male**	**12.51**	0.84	5	6.01	0.45	5	2.51	0.17	5
**Asphyxia**	**Male**	**9.91**	0.49	5	5.09	0.56	5	1.73	0.11	5
**Creatine**	**Male**	**10.03**	0.86	5	6.01	0.48	5	1.94	0.23	5
**Cr+Asphyxia**	**Male**	**11.15**	0.44	5	5.02	0.31	5	1.75	0.22	5
**C-Section**	**Female**	**10.66**	1.40	5	4.97	0.37	5	2.13	0.15	5
**Asphyxia**	**Female**	**9.58**	1.19	5	4.95	1.06	5	1.88	0.55	5
**Creatine**	**Female**	**12.98**	0.90	5	6.55	0.83	5	2.15	0.34	5
**Cr+Asphyxia**	**Female**	**11.61**	0.45	5	5.88	0.28	5	2.23	0.28	5
