# Supplementary material for: Maternal Creatine Supplementation during Pregnancy Prevents Long-Term Changes in Diaphragm Muscle Structure and Function after Birth Asphyxia
Source: PLoS One. 2016 Mar 1;11(3):e0149840. doi: 10.1371/journal.pone.0149840 (PMC4773130; doi:10.1371/journal.pone.0149840)
Supplement: S4 Table — (PDF) [file pone.0149840.s004.pdf]

		C-Section			Asphyxia			Creatine			Cr+Asphyxia		
	**Freq (Hz)**	**Mean**	**SEM**	**N**	**Mean**	**SEM**	**N**	**Mean**	**SEM**	**N**	**Mean**	**SEM**	**N**
**Male**	**2**	0.3	0.058	5	0.32	0.042	5	0.36	0.056	5	0.29	0.05	5
	**5**	0.38	0.042	5	0.41	0.058	5	0.42	0.035	5	0.39	0.042	5
	**10**	0.54	0.038	5	0.52	0.029	5	0.49	0.034	5	0.5	0.036	5
	**15**	0.67	0.029	5	0.65	0.037	5	0.62	0.028	5	0.63	0.035	5
	**20**	0.83	0.039	5	0.81	0.041	5	0.78	0.039	5	0.85	0.026	5
	**40**	0.92	0.025	5	0.91	0.029	5	0.89	0.024	5	0.89	0.031	5
	**60**	0.94	0.015	5	0.95	0.02	5	0.96	0.029	5	0.95	0.038	5
	**70**	1	0.01	5	1	0.019	5	1	0.022	5	1	0.018	5

		C-Section			Asphyxia			Creatine			Cr+Asphyxia		
	**Freq (Hz)**	**Mean**	**SEM**	**N**	**Mean**	**SEM**	**N**	**Mean**	**SEM**	**N**	**Mean**	**SEM**	**N**
**Female**	**2**	0.28	0.057	5	0.31	0.041	5	0.35	0.042	5	0.32	0.058	5
	**5**	0.35	0.043	5	0.39	0.056	5	0.41	0.036	5	0.41	0.042	5
	**10**	0.56	0.039	5	0.51	0.027	5	0.53	0.041	5	0.52	0.038	5
	**15**	0.65	0.028	5	0.66	0.036	5	0.61	0.031	5	0.65	0.029	5
	**20**	0.86	0.04	5	0.78	0.042	5	0.77	0.038	5	0.81	0.039	5
	**40**	0.9	0.024	5	0.91	0.031	5	0.92	0.035	5	0.91	0.025	5
	**60**	0.96	0.016	5	0.94	0.022	5	0.95	0.021	5	0.95	0.015	5
	**70**	1	0.012	5	1	0.018	5	1	0.022	5	1	0.01	5
