# Supplementary material for: Maternal Creatine Supplementation during Pregnancy Prevents Long-Term Changes in Diaphragm Muscle Structure and Function after Birth Asphyxia
Source: PLoS One. 2016 Mar 1;11(3):e0149840. doi: 10.1371/journal.pone.0149840 (PMC4773130; doi:10.1371/journal.pone.0149840)
Supplement: S5 Table — (PDF) [file pone.0149840.s005.pdf]

	C-Section		
	**Mean**	**SEM**	**N**
**Male**	20.57	2.91	5
**Female**	17.90	2.39	5

	Asphyxia		
	**Mean**	**SEM**	**N**
**Male**	12.25	2.65	5
**Female**	17.57	4.34	5

	Creatine		
	**Mean**	**SEM**	**N**
**Male**	25.95	5.05	5
**Female**	23.65	3.86	5

	Cr+Asphyxia		
	**Mean**	**SEM**	**N**
**Male**	24.35	2.69	5
**Female**	16.98	3.49	5
