# Supplementary material for: Maternal Creatine Supplementation during Pregnancy Prevents Long-Term Changes in Diaphragm Muscle Structure and Function after Birth Asphyxia
Source: PLoS One. 2016 Mar 1;11(3):e0149840. doi: 10.1371/journal.pone.0149840 (PMC4773130; doi:10.1371/journal.pone.0149840)
Supplement: S6 Table — (PDF) [file pone.0149840.s006.pdf]

	C-Section			Asphyxia			Creatine			Cr+Asphyxia		
	**Mean**	**SEM**	**N**	**Mean**	**SEM**	**N**	**Mean**	**SEM**	**N**	**Mean**	**SEM**	**N**
**Male**	0.79	0.11	5	0.51	0.03	5	0.80	0.05	5	0.64	0.02	5
**Female**	0.75	0.05	5	0.49	0.05	5	0.67	0.05	5	0.66	0.04	5

	C-Section				Asphyxia				Creatine				Cr+Asphyxia		
	**Mean**	**SEM**	**N**		**Mean**	**SEM**	**N**		**Mean**	**SEM**	**N**		**Mean**	**SEM**	**N**
**Male**	0.34	0.04	5	**Male**	0.18	0.03	5	**Male**	0.34	0.05	5	**Male**	0.30	0.02	5
**Female**	0.29	0.03	5	**Female**	0.22	0.04	5	**Female**	0.29	0.04	5	**Female**	0.25	0.02	5

	C-Section				Asphyxia				Creatine				Cr+Asphyxia		
	**Mean**	**SEM**	**N**		**Mean**	**SEM**	**N**		**Mean**	**SEM**	**N**		**Mean**	**SEM**	**N**
**Male**	20.57	2.91	5	**Male**	12.25	2.65	5	**Male**	25.95	5.05	5	**Male**	24.35	2.69	5
**Female**	17.90	2.39	5	**Female**	17.57	4.34	5	**Female**	23.65	3.86	5	**Female**	16.98	3.49	5

	C-Section			
	Contraction	Mean	SEM	N
Male	1	100.00	0.00	5
	50	78.82	10.58	5
	100	57.23	7.05	5
	150	44.35	5.85	5
	200	41.11	5.45	5
	250	35.99	4.75	5
	300	33.84	3.97	5
		Mean	SEM	N
Female	1	100.00	0.00	5
	50	68.38	6.49	5
	100	49.00	4.10	5
	150	39.61	4.89	5
	200	33.24	5.17	5
	250	27.98	4.04	5
	300	25.96	4.04	5

	Asphyxia			
	Contraction	Mean	SEM	N
Male	1	100.00	0.00	5
	50	50.67	3.23	5
	100	40.48	2.94	5
	150	33.65	3.00	5
	200	28.92	2.92	5
	250	21.11	2.54	5
	300	18.20	2.69	5
		Mean	SEM	N
Female	1	100.00	0.00	5
	50	60.00	6.58	5
	100	43.55	6.30	5
	150	36.47	5.66	5
	200	30.66	4.89	5
	250	26.48	4.63	5
	300	22.88	4.33	5

	Creatine			
	Contraction	Mean	SEM	N
Male	1	100.00	0.00	5
	50	79.82	4.71	5
	100	57.51	5.06	5
	150	51.14	4.77	5
	200	47.15	4.84	5
	250	42.60	5.50	5
	300	38.86	6.02	5
		Mean	SEM	N
Female	1	100.00	0.00	5
	50	67.36	5.22	5
	100	48.03	4.61	5
	150	41.40	3.75	5
	200	35.45	3.47	5
	250	32.00	3.67	5
	300	28.41	3.54	5

	Cr+Asphyxia			
	Contraction	Mean	SEM	N
Male	1	100.00	0.00	5
	50	66.66	2.43	5
	100	44.43	4.53	5
	150	39.57	4.53	5
	200	35.81	4.48	5
	250	31.23	2.41	5
	300	26.87	2.44	5
		Mean	SEM	N
Female	1	100.00	0.00	5
	50	65.54	5.17	5
	100	46.43	4.51	5
	150	38.34	4.18	5
	200	32.55	4.66	5
	250	27.15	4.56	5
	300	21.59	4.05	5

		C-Section			Asphyxia			Creatine			Cr+Asphyxia		
	**Freq (Hz)**	**Mean**	**SEM**	**N**	**Mean**	**SEM**	**N**	**Mean**	**SEM**	**N**	**Mean**	**SEM**	**N**
**Male**	**2**	0.3	0.058	5	0.32	0.042	5	0.36	0.056	5	0.29	0.05	5
	**5**	0.38	0.042	5	0.41	0.058	5	0.42	0.035	5	0.39	0.042	5
	**10**	0.54	0.038	5	0.52	0.029	5	0.49	0.034	5	0.5	0.036	5
	**15**	0.67	0.029	5	0.65	0.037	5	0.62	0.028	5	0.63	0.035	5
	**20**	0.83	0.039	5	0.81	0.041	5	0.78	0.039	5	0.85	0.026	5
	**40**	0.92	0.025	5	0.91	0.029	5	0.89	0.024	5	0.89	0.031	5
	**60**	0.94	0.015	5	0.95	0.02	5	0.96	0.029	5	0.95	0.038	5
	**70**	1	0.01	5	1	0.019	5	1	0.022	5	1	0.018	5

		C-Section			Asphyxia			Creatine			Cr+Asphyxia		
	**Freq (Hz)**	**Mean**	**SEM**	**N**	**Mean**	**SEM**	**N**	**Mean**	**SEM**	**N**	**Mean**	**SEM**	**N**
**Female**	**2**	0.28	0.057	5	0.31	0.041	5	0.35	0.042	5	0.32	0.058	5
	**5**	0.35	0.043	5	0.39	0.056	5	0.41	0.036	5	0.41	0.042	5
	**10**	0.56	0.039	5	0.51	0.027	5	0.53	0.041	5	0.52	0.038	5
	**15**	0.65	0.028	5	0.66	0.036	5	0.61	0.031	5	0.65	0.029	5
	**20**	0.86	0.04	5	0.78	0.042	5	0.77	0.038	5	0.81	0.039	5
	**40**	0.9	0.024	5	0.91	0.031	5	0.92	0.035	5	0.91	0.025	5
	**60**	0.96	0.016	5	0.94	0.022	5	0.95	0.021	5	0.95	0.015	5
	**70**	1	0.012	5	1	0.018	5	1	0.022	5	1	0.01	5
