# Supplementary material for: Maternal Creatine Supplementation during Pregnancy Prevents Long-Term Changes in Diaphragm Muscle Structure and Function after Birth Asphyxia
Source: PLoS One. 2016 Mar 1;11(3):e0149840. doi: 10.1371/journal.pone.0149840 (PMC4773130; doi:10.1371/journal.pone.0149840)
Supplement: S7 Table — (PDF) [file pone.0149840.s007.pdf]

	C-Section				Asphyxia				Creatine				Cr+Asphyxia	
	**Mean**	**SEM**	**N**		**Mean**	**SEM**	**N**		**Mean**	**SEM**	**N**		**Mean**	**SEM**	**N**
**Male**	0.34	0.04	5	**Male**	0.18	0.03	5	**Male**	0.34	0.05	5	**Male**	0.30	0.02	5
**Female**	0.29	0.03	5	**Female**	0.22	0.04	5	**Female**	0.29	0.04	5	**Female**	0.25	0.02	5
