# Supplementary material for: Maternal Creatine Supplementation during Pregnancy Prevents Long-Term Changes in Diaphragm Muscle Structure and Function after Birth Asphyxia
Source: PLoS One. 2016 Mar 1;11(3):e0149840. doi: 10.1371/journal.pone.0149840 (PMC4773130; doi:10.1371/journal.pone.0149840)
Supplement: S8 Table — (PDF) [file pone.0149840.s008.pdf]

	C-Section			
	Contraction	Mean	SEM	N
Male	**1**	100.00	0.00	5
	**50**	78.82	10.58	5
	**100**	57.23	7.05	5
	**150**	44.35	5.85	5
	**200**	41.11	5.45	5
	**250**	35.99	4.75	5
	**300**	33.84	3.97	5
		Mean	SEM	N
Female	**1**	100.00	0.00	5
	**50**	68.38	6.49	5
	**100**	49.00	4.10	5
	**150**	39.61	4.89	5
	**200**	33.24	5.17	5
	**250**	27.98	4.04	5
	**300**	25.96	4.04	5

	Asphyxia			
	Contraction	Mean	SEM	N
Male	**1**	100.00	0.00	5
	**50**	50.67	3.23	5
	**100**	40.48	2.94	5
	**150**	33.65	3.00	5
	**200**	28.92	2.92	5
	**250**	21.11	2.54	5
	**300**	18.20	2.69	5
		Mean	SEM	N
Female	**1**	100.00	0.00	5
	**50**	60.00	6.58	5
	**100**	43.55	6.30	5
	**150**	36.47	5.66	5
	**200**	30.66	4.89	5
	**250**	26.48	4.63	5
	**300**	22.88	4.33	5

	Creatine			
	Contraction	Mean	SEM	N
Male	**1**	100.00	0.00	5
	**50**	79.82	4.71	5
	**100**	57.51	5.06	5
	**150**	51.14	4.77	5
	**200**	47.15	4.84	5
	**250**	42.60	5.50	5
	**300**	38.86	6.02	5
		Mean	SEM	N
Female	**1**	100.00	0.00	5
	**50**	67.36	5.22	5
	**100**	48.03	4.61	5
	**150**	41.40	3.75	5
	**200**	35.45	3.47	5
	**250**	32.00	3.67	5
	**300**	28.41	3.54	5

	Cr+Asphyxia			
	Contraction	Mean	SEM	N
Male	**1**	100.00	0.00	5
	**50**	66.66	2.43	5
	**100**	44.43	4.53	5
	**150**	39.57	4.53	5
	**200**	35.81	4.48	5
	**250**	31.23	2.41	5
	**300**	26.87	2.44	5
		Mean	SEM	N
Female	**1**	100.00	0.00	5
	**50**	65.54	5.17	5
	**100**	46.43	4.51	5
	**150**	38.34	4.18	5
	**200**	32.55	4.66	5
	**250**	27.15	4.56	5
	**300**	21.59	4.05	5
